# Evaluating the Cost-Effectiveness of Cervical Cancer Screening and Treatment in Western Romania

**DOI:** 10.3390/curroncol32060336

**Published:** 2025-06-07

**Authors:** Ion Petre, Șerban Mircea Negru, Florina Buleu, Radu Dumitru Moleriu, Marina Adriana Mercioni, Izabella Petre, Anca Bordianu, Vladiana Turi, Luciana Marc, Daian Ionel Popa, Daliborca Cristina Vlad

**Affiliations:** 1 Doctoral School, “Victor Babes” University of Medicine and Pharmacy, 300041 Timisoara, Romania; petre.ion@umft.ro (I.P.); daian-ionel.popa@umft.ro (D.I.P.); 2 Department of Functional Sciences, Medical Informatics and Biostatistics Discipline, “Victor Babes” University of Medicine and Pharmacy, 300041 Timisoara, Romania; radu.moleriu@umft.ro; 3 Department of Oncology, “Victor Babes” University of Medicine and Pharmacy of Timisoara, 300041 Timisoara, Romania; serban.negru@umft.ro; 4 Department of Cardiology, “Victor Babes” University of Medicine and Pharmacy Timisoara, 300041 Timisoara, Romania; 5 “Victor Babes” University of Medicine and Pharmacy Timisoara, 300041 Timisoara, Romania; 6 Applied Electronics Department, Faculty of Electronics, Telecommunications and Information Technologies, Politehnica University Timișoara, 300223 Timisoara, Romania; 7 Department XII of Obstetrics and Gynaecology, “Victor Babes” University of Medicine and Pharmacy, 300041 Timisoara, Romania; petre.izabella@umft.ro; 8 Department of Plastic Surgery and Reconstructive Microsurgery “Bagdasar-Arseni” Emergency Hospital Bucharest, University of Medicine and Pharmacy “Carol Davila”, 010825 Bucharest, Romania; anca.bordianu@gmail.com; 9 Department of Internal Medicine, “Victor Babes” University of Medicine and Pharmacy Timisoara, 300041 Timisoara, Romania; turi.vladiana@umft.ro; 10 Department VII of Internal Medicine II, Division of Nephrology, “Victor Babes” University of Medicine and Pharmacy Timisoara, 300041 Timisoara, Romania; marc.luciana@umft.ro; 11 Research Center for Medical Communication, “Victor Babes” University of Medicine and Pharmacy, 300041 Timisoara, Romania; 12 Department of Biochemistry and Pharmacology, “Victor Babes” University of Medicine and Pharmacy Timisoara, 300041 Timisoara, Romania; vlad.daliborca@umft.ro

**Keywords:** cost-effectiveness, cervical cancer, treatment, surgery, cancer screening

## Abstract

**Background and Objectives:** As a leading European country in terms of cervical cancer incidence and mortality, there has been a pressing need for Romania to upgrade its cervical cancer management. The criteria set by the International Federation of Gynecology and Obstetrics indicate that different treatments should have a similar trend concerning progression-free survival and overall survival at all the various stages of cervical cancer. This study aimed to assess the cost-effectiveness (CE) of the primary treatment plans related to the survival rate for cervical cancer screening in the western part of Romania and provide some recommendations. **Materials and Methods:** Descriptive statistics and a correlation model were used to examine costs. AI models have been developed to forecast the CE of different treatments using the above-mentioned studies on overall survival rates and treatment-related toxicity rates for five years. The costs of cervical cancer treatment were sourced from the public health department, the oncology clinic in the western region of Romania, and the County Hospital available for each stage. **Results:** Treatment expenses vary by cancer stage, with a significant increase from stages IA/IB to IIA, stabilizing between IIA and IIIC (about €7800–€8300), followed by a steep decline in IVA and a more pronounced decrease in IVB and in situ. The results highlight certain treatment combinations and their costs, indicating that the highest costs (exceeding €8000) are linked to multimodal treatments, which encompass surgery, chemotherapy, radiotherapy, and brachytherapy. **Conclusions**: Advanced cancer stages (IIA–IIIC) entail the highest treatment costs due to intricate, multimodal therapy, whereas early stages (IA, IB, in situ) and late terminal stages (IVB) are linked to considerably reduced treatment costs.

## 1. Introduction

Hospitals are using an increasing amount of resources allocated to the healthcare system, although the quality of treatment is improving and utilization rates are consistently being maintained. In Romania, public hospital spending is higher than in other European nations, which is counterintuitive given the country’s low wages. There are apparent political and societal hazards in this case. Furthermore, it calls into question primary care’s position as the focus of change. Romania continues to encounter certain obstacles, such as wealth disparity and restricted access to healthcare and education in some regions. The nation also struggles with environmental issues, such as pollution of the air and water [1,2,3].

The choice between surgery and adjuvant chemoradiotherapy ultimately comes down to competing considerations such as quality of life, treatment toxicity, cancer control (survival), and patient location regarding available healthcare resources [4]. Adjuvant chemotherapy and hormone therapy significantly impacted treatment expenses per patient [5,6].

In recent years, there has been a growing trend in the expenditures associated with cancer screening and treatment. Chemotherapy costs are the most significant expense [7].

In Romania, the majority of the funding for the health system comes from public sources (80.45%), including Social Health Insurance (65%) and the State and Local Authorities Budget (15.45%). An additional 19.55% comes from private sources, including voluntary health insurance and out-of-pocket expenses. The sector’s significance in the overall health system is evident from the proportions of the various expenditure categories, and patterns in spending demonstrate how finance affects structural changes in the health sector [8].

In the European Union, there are wide variations in the prevalence of cervical cancer. Although incidence has decreased recently, especially for those under 65, Romania has historically had some of the highest rates of female cervical cancer mortality in the EU [9]. This has been linked to inadequate coverage and quality of screening practices and a lack of systematic screening. Screening for cervical cancer and treating identified lesions can significantly reduce the death rate from the disease [10].

Compared to Finland, the nation in Europe now experiencing the lowest cervical cancer burden, Romania had an incidence and death rate from cervical cancer that are, respectively, around five and twelve times higher. Cervical cancer is currently a gynecological cancer linked to the most significant incidence and death in Eastern Europe [11].

Comprehensive, multimodal treatments, which may combine surgery, radiation, and chemotherapy, are frequently used to treat women with newly diagnosed cervical cancer. The price of medical care and how it is billed to patients are not well known [12].

Human papillomavirus (HPV) infections cause about 5% of all cancers, which includes malignancies in the penis, vulva, vagina, anus, oropharynx, and, most commonly, the cervix. The most carcinogenic HPV is HPV-16, which is the dominant cancer-causing type. There is also strong evidence that HPV-18, -31, -33, -35, -39, -45, -51, -52, -56, -58, and -59 can cause cervical cancer. All commercial HPV vaccines are composed of the L1 protein of this open reading frame, which is capable of forming VLPs and may lead then to HPV type-specific neutralizing antibodies. A total of six prophylactic HPV vaccines have been licensed, consisting of three bivalent, two quadrivalent, and one nonavalent. The bivalent vaccines target HPV-16 and -18, which account for more than 70% of all cervical cancers [13]. HPV type 18, which was previously a major contributor to cervical cancer, has experienced a notable reduction in prevalence among populations that have been vaccinated. Research conducted in Germany indicated that HPV type 18 represented merely 0.5% of high-risk HPV infections, positioning it fifth among other strains such as HPV-16, -52, and -31 [14].

Over the past decades, the distribution of HPV types has shifted, contributing to changes in the prevalence and progression patterns of cervical lesions, including low-grade squamous intraepithelial lesions (LSIL—CIN 1) and high-grade squamous intraepithelial lesions (HSIL—CIN 2/3) [15]. A research investigation into the effects of HPV vaccination in Denmark revealed a notable reduction in the rates of cervical intraepithelial neoplasia grade 2 (CIN2) and cervical cancer during the timeframe subsequent to the implementation of the second catch-up HPV vaccination initiative (2013–2019), with the most pronounced decline recorded among women aged 30 and below [16].

In countries with sufficient funds, cervical cancer prevention methods that combine the human papillomavirus 16/18 vaccine with HPV screening are highly efficient. Low vaccine costs are necessary for combination immunization and screening procedures to be financially viable in low-resource environments [17].

When treating early-stage, high-risk cervical cancer, participants consistently favored surgical excision and opted for the least intrusive procedure. Using such utility scores, quality of life impacts may be included in comparative–effectiveness models for cervical cancer [18]. Currently, the best treatments for early-stage cervical cancer are hysterectomy and pelvic lymph node dissection. More adjuvant therapy can be considered if there is a chance of a recurrence. For individuals with high-risk characteristics, postoperative pelvic irradiation combined with concurrent platinum-based chemotherapy is advised [19]. Adjuvant hysterectomy may improve the prognosis for patients with bulky cervical carcinoma whose brachytherapy doses were not administered appropriately [20].

With no increase in toxicity, postoperative adjuvant chemoradiotherapy (CRT) can help individuals with intermediate risk factors for cervical cancer [21]. Radiotherapy was thought to be less expensive than chemoradiation. Furthermore, chemoradiation was more successful than radiation therapy [22]. Extremely affordable strategies available to fight cervical cancer include screening using smear tests or visual inspection [23] in conjunction with treatment for cervical cancer [24]. In resource-constrained settings, radical radiotherapy would be the preferred treatment option for FIGO stage IIB cervical cancer since it is a more economical approach [25].

Chemotherapy significantly extends the average woman’s quality-adjusted life expectancy at a cost similar to other commonly acknowledged medicines. If long-term survival changes less than disease-free survival, this benefit diminishes significantly [26].

Of the women aged 30–49, two out of every three had never had a cervical cancer screening [27]. Screening uptake is extremely low in low- and middle-income nations, where the disease burden is most significant. Increasing screening coverage and treating lesions that are discovered should be the top priorities of the WHO elimination campaign. However, increasing surveillance system efforts regarding coverage and quality control will be a significant obstacle to reaching the WHO eradication objective [28].

It has been demonstrated that having several sexual partners and starting sexual relations early significantly increases the risk. The adoption of screening impacts the significant variations in incidence among nations. There are indications of an increasing risk of cervical cancer, most likely because of changes in sexual behavior, even if the overall picture of incidence and death is still low. Human papillomavirus (HPV) type 16/18 and smoking are presently significant factors in the multifactorial, stepwise carcinogenesis concept in the cervix uteri. As a result, screening programs, HPV vaccination campaigns, and society-based preventative and control measures should be implemented. Molecular testing has replaced cell morphology inspection as the primary way of screening for cervical cancer. Liquid-based cytology and high-risk HPV genotyping are standard techniques extensively advised and applied globally [29].

Given its improved clinical performance and decreased referral rate, machine learning has the potential to be a viable screening option for cervical cancer. It can also construct models based on the present screening markers for the disease [30].

Cervical cancer is still a significant health issue in the area; thus, efforts should be made to remove obstacles by putting a four-step plan for the eradication of cervical cancer in Europe into action. Four critical areas of evidence-based interventions—vaccination, screening, treatment, and public awareness—can help achieve this aim [31]. One of the screening techniques used to find cervical cancer is the Papanicolaou Test, often known as the Pap smear test. The primary benefit that it offers is its affordability [32,33].

According to current guidelines, adjuvant radiotherapy for early-stage cervical cancer should be considered if several risk indicators, including tumor size, lymphovascular space invasion, and depth of stromal invasion, are in the diagnostic phase [34].

The cost of HPV vaccination is a significant obstacle to its fair distribution in low-income nations, where health resources are constrained and must balance competing demands [35]. About 80% of cervical cancer fatalities worldwide occur in underdeveloped nations, accounting for an estimated 242,000 deaths as opposed to 33,000 deaths in high-income countries. In underdeveloped countries, HPV vaccination would seem to be the natural measure to minimize cervical cancer mortality, given the gaps in secondary prevention [36].

It is acknowledged that cervical cancer is caused by HPV infection, and there is mounting evidence that HPV may also play a role in other anogenital malignancies, including head and neck cancers as well as in the anus, vulva, vagina, and penis. Worldwide, HPV types 16 and 18 account for over 70% of cases of cervical cancer. Cervical and other anogenital malignancies may become less familiar with the introduction of HPV vaccinations that guard against HPV 16 and 18. Around 2972.8 million women worldwide who are at least 15 years of age are susceptible to cervical cancer [37]. The highest age-standardized incidence rates, according to the WHO, were registered in Romania, Montenegro, Serbia, Lithuania, Estonia, Latvia, Bulgaria, and Hungary [38].

As technology is used increasingly in the medical industry, artificial intelligence (AI) has become a viable tool for raising diagnostic precision and lowering errors. AI can swiftly examine vast volumes of medical data, including tabular data, and detect anomalies a person would overlook. AI can identify patterns and abnormalities in medical data by using those techniques, which enables it to diagnose patients more quickly, accurately, and efficiently or to determine if a particular profile is susceptible to developing a specific disease [39,40,41,42,43].

AI applications in medical diagnostics have grown in popularity recently and are very applicable to detecting and diagnosing cervical cancer. They have the advantage of requiring less time, less technical and professional staff, and not being biased due to subjective variables [44,45,46,47,48,49,50,51,52,53,54,55,56].

The main objectives of this article are to predict the cost based on the available data on cervical cancer screening in Romania, to identify a feasible and cost-effective screening strategy for low-resource settings, and to foresee the challenges in implementing effective cervical cancer screening.

## 2. Materials and Methods

The World Health Organization (WHO) recognizes two major histological types of invasive cancer:

- Squamous carcinoma (which accounts for about 85% of all cases).
- Adenocarcinoma (which accounts for about 10–15% of all cases).

Other carcinomas include adenosine carcinomas, cystic adenoid carcinoma, neuroendocrine tumors, mesenchymal tumors, mixed epithelial/mesenchymal tumors, and metastatic carcinoma, which comprise 3–5% of all cases.

The American Cancer Society has updated its screening recommendations, which state that women with cervixes older than 25 should get screened for cervical cancer. Every five years, women between the ages of 25 and 65 should have their first HPV test completed. For other anomalies, rescreening is advised after a year, and for normal ones, after three years [57]. Population screening enables early diagnosis and treatment due to the extended natural history and protracted precancerous phase. Invasive cervical cancer is still the most prevalent neoplasia in women in several countries and the fourth most common malignancy in women worldwide, despite screening [58]. Cervical cancer may result from a persistent high-risk HPV infection [59].

The data was collected from 2018 to 2022, and the clinical features of patients with cervical cancer who received different treatments according to their stage were recorded using medical histories. Furthermore, the expenses for each patient were determined based on how their illness was progressing. Analysis was limited to patients who completed an informed consent form and supplied complete data. The Public Health Department (DSP) and the oncological clinic Oncohelp Timisoara have assured the availability of data. The DSP data contains three features: year, urban, and rural cancer cases.

**Figure 1:**
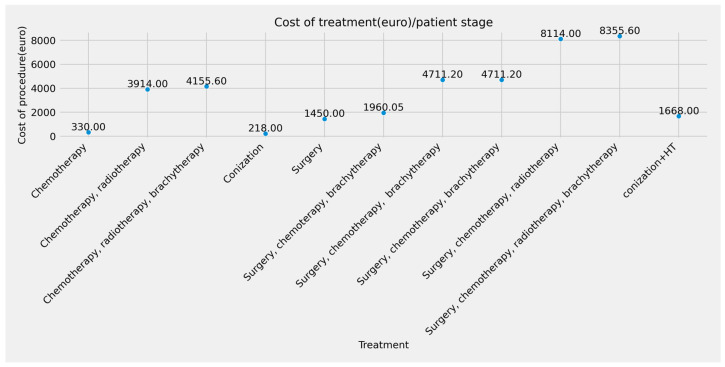
Cervical cancer costs according to the treatment type.

The Oncohelp data include more variables, such as date, age, diagnosis, and stage (Figure 1). For each stage, we calculated the cost (€) feature, which displays the total amount spent on each patient based on their stage of cervical cancer.

The research approach combines studies and descriptive statistics with artificial intelligence analysis and prediction, considering the inter- and transdisciplinary techniques required to address this challenging problem, involving quantitative and qualitative methods to estimate the cost of reducing cancer spread and cost efficacy.

The JASP program (version 2023) was used for the statistical analysis. We conducted descriptive statistics and calculated the cost distribution based on the stage and therapy used to obtain the results for the discussed instances. The confidence level was set at α = 0.05. For the statistical analysis, descriptive statistics were made, and the Mann–Whiteney and Kruskal–Wallis tests were applied.

### The Artificial Intelligence Algorithms

Compared to traditional regression models, AI models—especially those that use machine learning—can analyze huge, complicated datasets more quickly and correctly. With the use of this ability, subtle patterns and linkages that conventional statistical methods would overlook might be found, possibly producing more accurate forecasts and more insightful health economic analyses.

A wider range of data sources, such as wearable technology, genomics, and electronic health records, are becoming more and more important in health economics. While classical time series regression could have trouble with scalability and non-linear correlations, artificial intelligence (AI) is excellent at integrating and learning from such diverse, high-dimensional data [60].

If AI models are not appropriately trained or verified across a variety of groups, they may occasionally make health inequities worse. Furthermore, if patient demographics and clinical procedures change over time, their performance can deteriorate, requiring constant observation and adjustment [61].

**Figure 2:**
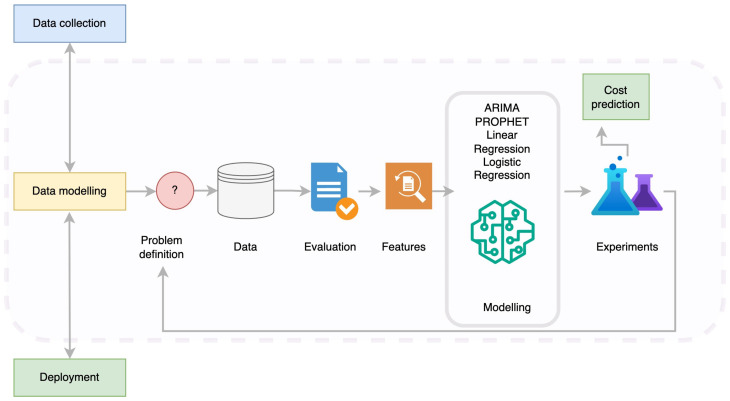
The architecture proposed to detect the evolution of costs.

As for methods, we used more methods to forecast time series data: Prophet and autoregressive integrated moving average (ARIMA) [62,63] and linear regression [64]. *Prophet* is an open-source software (Core Data Science team of Facebook) that involves fitting non-linear trends with annual, monthly, and daily seasonality and holiday impacts using an additive model. Time series with significant seasonal effects and several seasons of historical data perform the best. We decided to use Prophet because it manages outliers effectively and is resilient to missing data and trends. Both models are fitted to time series data to better understand the data or predict future series points. ARIMA models are used when data exhibit non-stationarity in mean (but not variance or autocovariance). In these situations, the non-stationarity of the mean function can be eliminated by applying an initial differencing step one or more times, corresponding to the “integrated” part of the model. We propose a binary target variable based on *Cost* feature, *Is_High_Cost*-1 if cost > median, 0 otherwise, and trained a Logistic Regression classifier. Figure 2 depicts the architecture proposed to detect the evolution of the cost spent on cancer cervical combat. The data was split into training (2985 patients—80%) and testing (747 patients—20%) sets. We used metrics like R-squared, Mean Absolute Error (MAE), Mean Squared Error (MSE), and Root Mean Squared Error (RMSE) [65] to evaluate linear regression model.

## 3. Results

Cost-effectiveness analysis is a method for estimating the socio-economic impact of adding more therapeutic options—both in terms of cost reduction—and AI modeling methods have been employed for this purpose.

### 3.1. Data Analysis and Statistics

**Figure 3:**
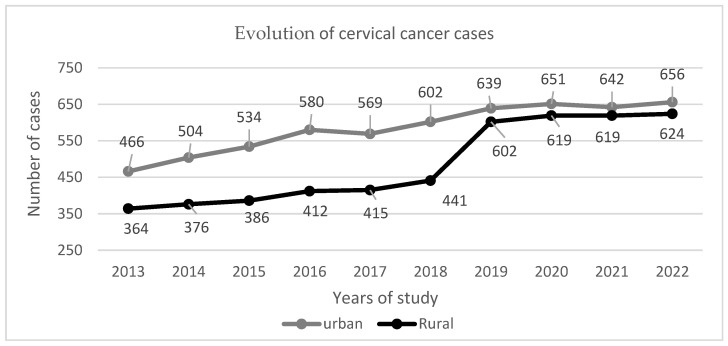
Cervical cancer cases declared by DSP yearly in urban and rural environments.

**Figure 4:**
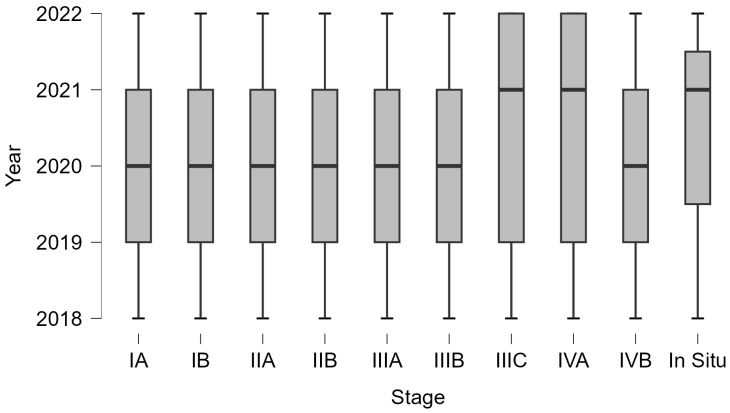
Cost incidence. Using a boxplot graphical representation, it can be seen that the cost has an increasing trend.

Figure 3 and Figure 4 displays cervical cancer records from the previous years (from 2013 to 2022) broken down by origin (rural, urban). This indicates a rising tendency; instances nearly doubled in less than a decade.

**Figure 5:**
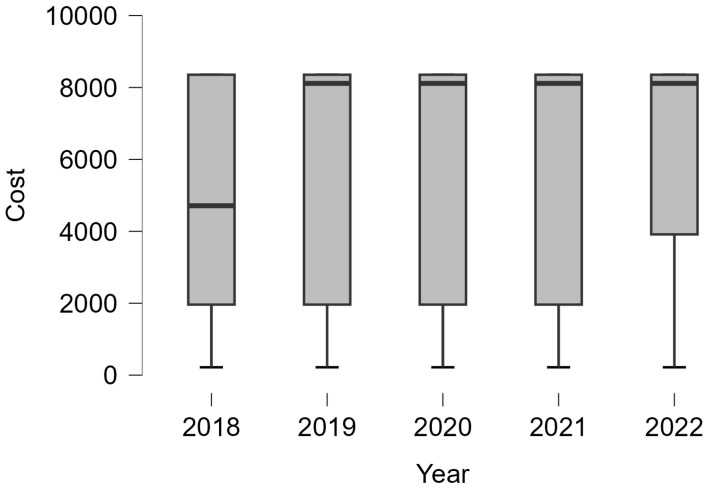
Staging cervical cancer incidence.

**Figure 6:**
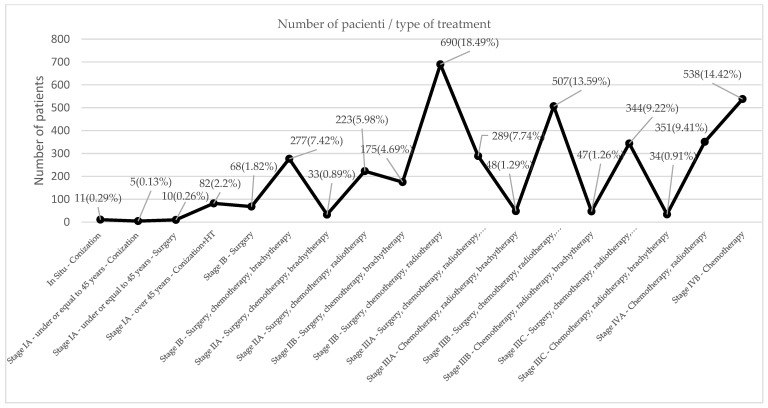
Staging cervical cancer cases declared by Oncohelp yearly.

A massive increase in all stages is seen in 2022, resulting in high costs. Decisions must be made to save patients’ lives and lower the cost of their treatments. Table 1 lists the expenses incurred at each stage; the patients in IIB, IIIB, and IVB, who make up the bulk of cases, have the highest costs because the treatment of this type of case needs a more complex approach, which includes multiple treatments of adjuvant therapy: radiotherapy, chemotherapy, surgery, and brachytherapy. The patients in Table 2 with the most significant treatment expenses require radiotherapy, chemotherapy, surgery, and brachytherapy (Figure 5 and Figure 6).

Table 3 shows the patient’s age, which unequivocally indicates that steps beyond the actual legal range for Pap tests and screenings are required to prevent cervical cancer and to combat it.

**Figure 7:**
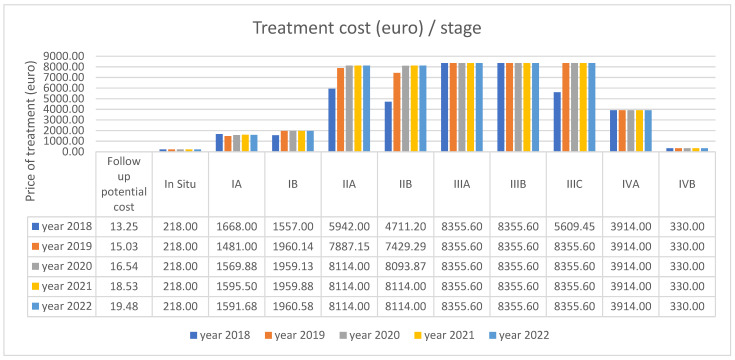
The average cost of each treatment/stage/year (euro) compared to a follow-up potential cost.

In Figure 7, a financial comparison between different stages of cervical cancer and costs is shown and how they change over five years (2018–2022).

**Figure 8:**
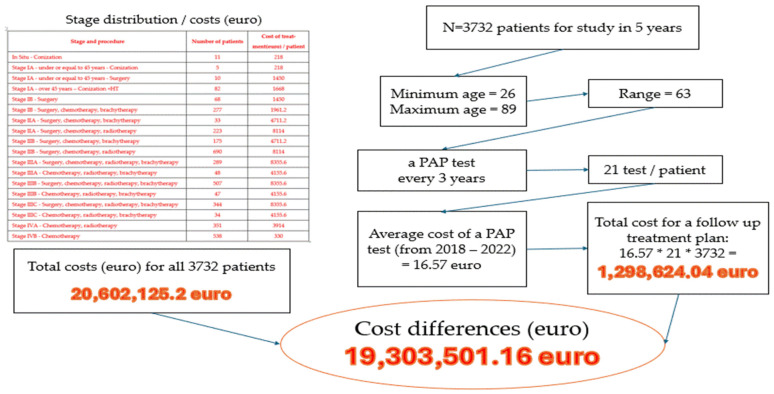
The significant differences registered between the costs that the state pays for the patient care compared to a follow-up treatment plan.

In Figure 8, the cost differences are explained thoroughly using the stage of disease, the total cost for a follow-up treatment plan, age, the average cost of a PAP smear, and the number of patients. Almost 20 million euros can be saved from the National Health Fund for 3732 patients if a PAP smear is performed every three years, thus preventing the disease from reaching an early stage.

A Mann–Whitney test was applied to determine the statistical significance of this study. For this, besides our group of 3732 patients, we included a control group of 5854 patients with the same characteristics, who in these five years went to two gynecological consultations (spending an average amount of 38.42 euro), which is significantly lower in cost than any treatment that we studied $\left(p<0.001\right)$.

**Figure 9:**
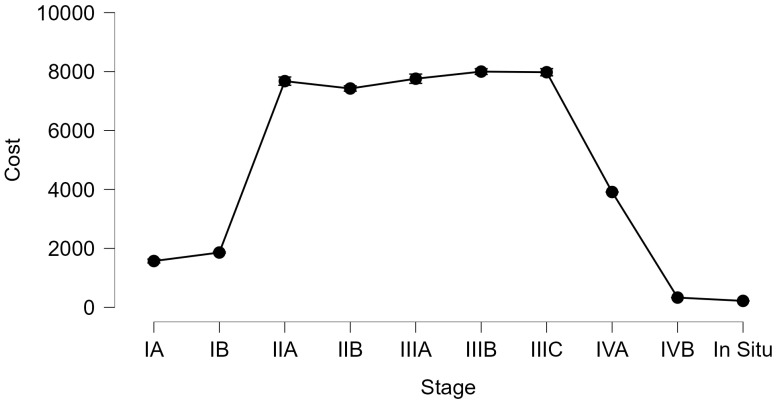
The cost–stage dependence.

Furthermore, a Kruskal–Wallis test was applied to see if there are significant differences between the costs that are involved in each stage, resulting in statistically significant differences $\left(p<0.001\right)$; the evolution cost of each treatment is presented in Figure 9.

**Figure 10:**
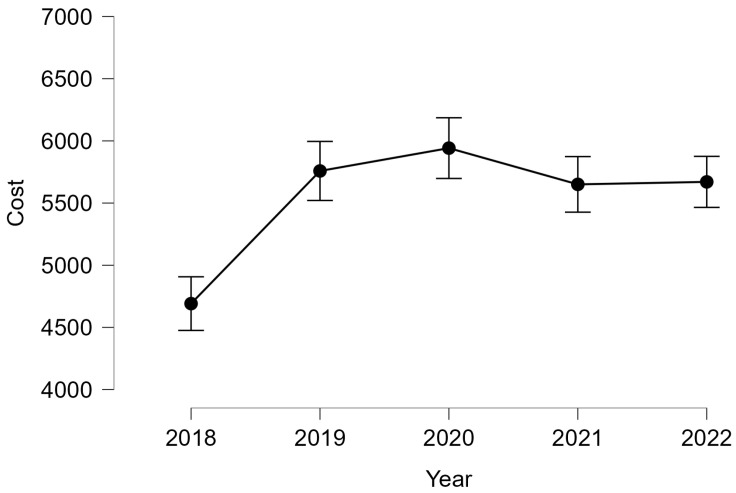
The cost–year dependence.

The evolution of costs during the studied years was tested, obtaining significant differences $\left(p<0.05\right)$, resulting from the Kruskal–Wallis test. The biggest costs were registered in 2020. The data evolution is plotted in Figure 10.

### 3.2. Artificial Intelligence

**Figure 11:**
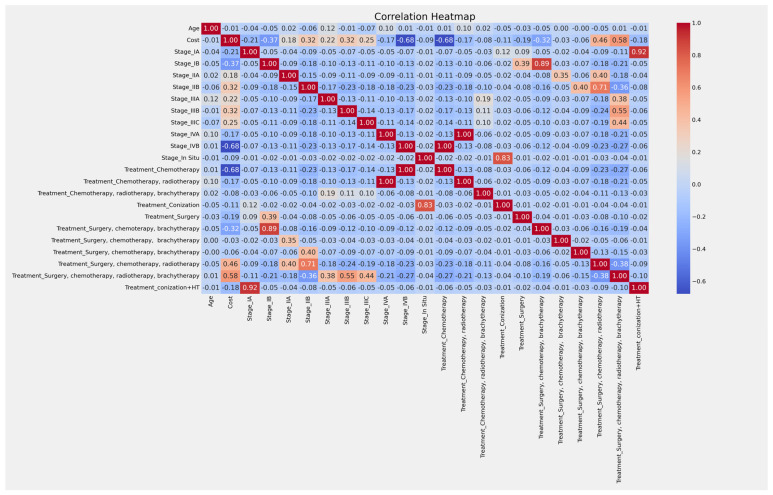
Confusion matrix of features correlation.

The main topics of this section are the forecast of costs using previous data and the consequences of not taking additional action. The data consists of 3732 recordings. The features correlation is displayed in Figure 11. The intense color shows a significant direct or indirect association between characteristics.

**Figure 12:**
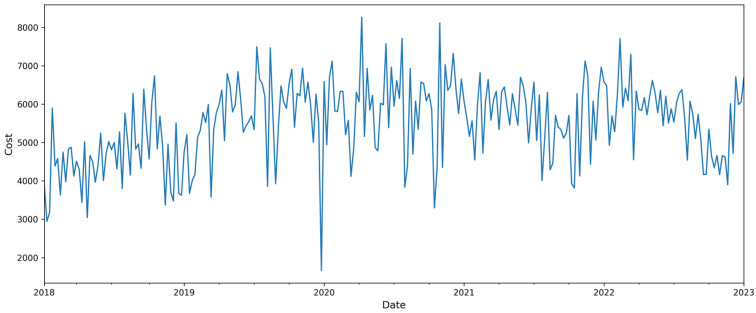
Data resampling to weekly.

**Figure 13:**
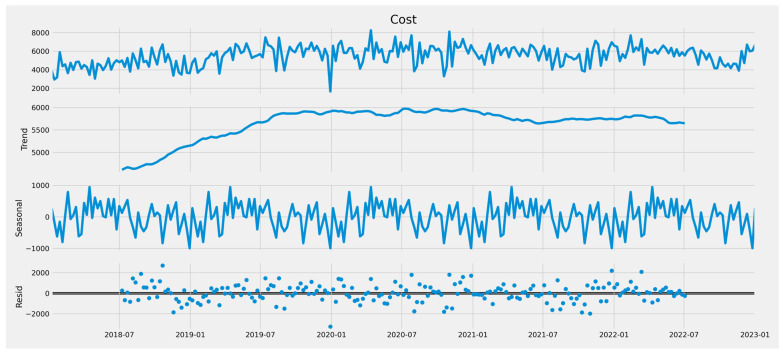
The seasonal decomposing data.

The cost data have been resampled, applying the mean weekly (Figure 12). The seasonal decomposing data using an additive model are shown in Figure 13.

**Figure 14:**
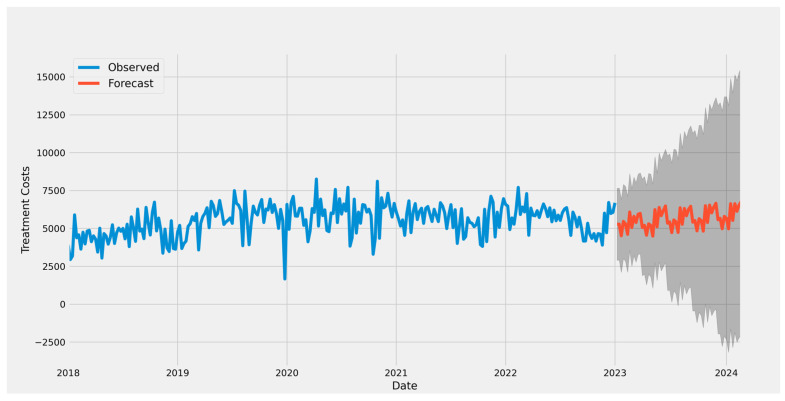
The costs are forecast using the ARIMA model.

**Figure 15:**
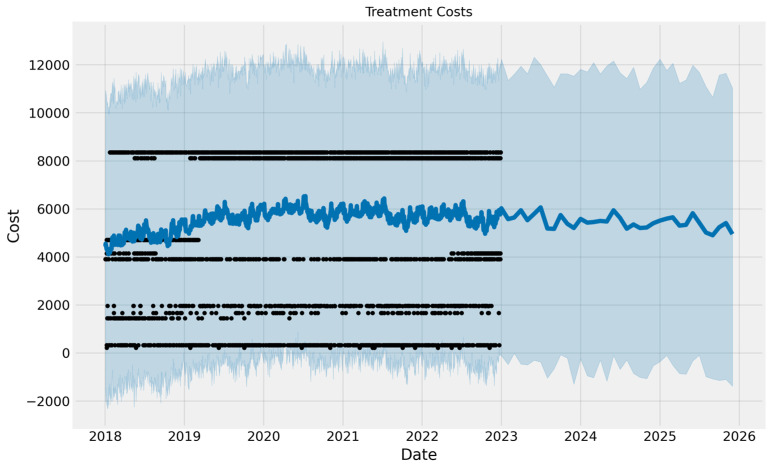
The costs are forecast using the Prophet model for the next three years.

The trend of costs is not that steady; it increases. Using the ARIMA model, we forecast costs for the next 96 weeks in advance (Figure 14). Because the Prophet algorithm accounts for outliers, we also employed it. Additionally, data points that differ from the overall dataset observations are eliminated. Seasonality and other impacts are handled by it. It manages the dataset’s spikes and incorporates them into the model’s training (Figure 15).

**Figure 16:**
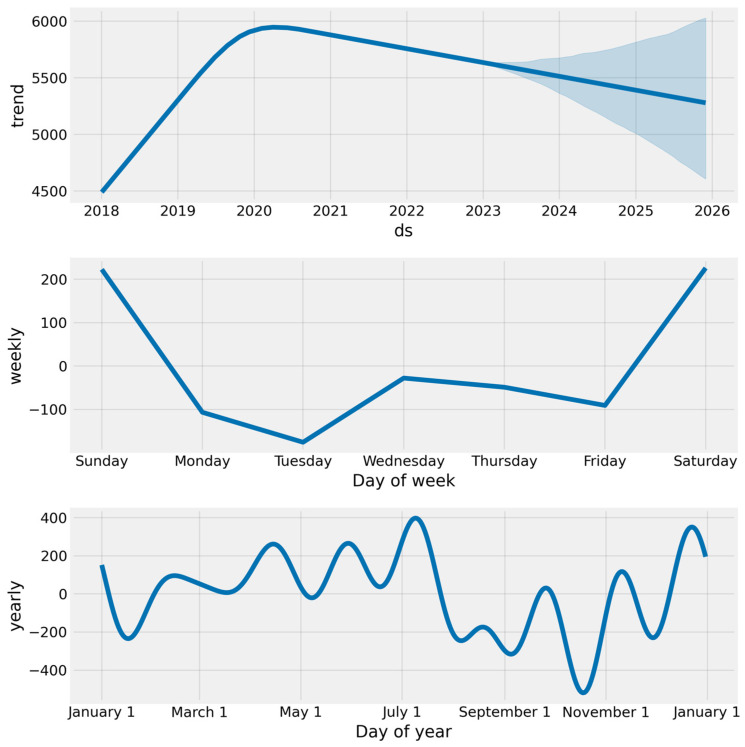
The costs are forecast using Prophet components.

In Figure 16, the output above depicts the trend and yearly seasonality components. The above plots provide insights into costs. The first plot shows a linear increase in cost from 2018 to the end of 2020. The next three years look to have a slight decrease in costs. The yearly plot shows that most incidence cases occur during July and January.

**Figure 17:**
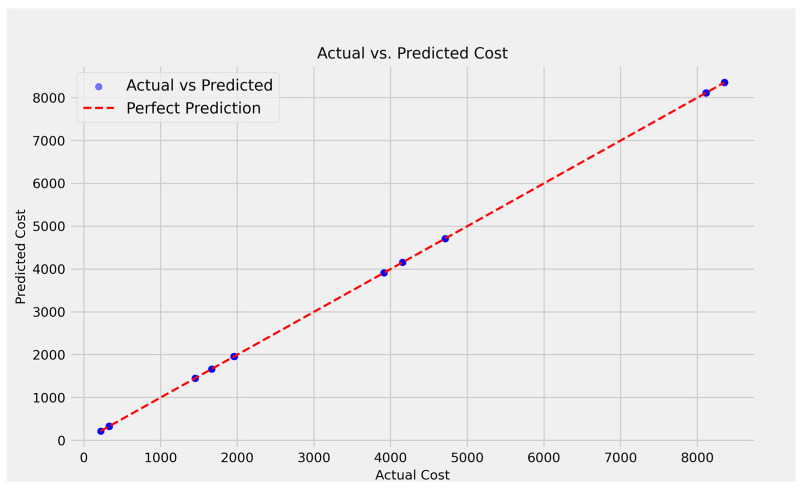
The predicted costs with the linear regression model.

The scatter plot of actual vs. predicted costs (Figure 17) demonstrates that the linear regression model performs well, with data points closely overlapping and the red dashed line signifying perfect prediction. This implies that the model’s predictions are very accurate, since the predicted values closely match the actual values throughout the cost range. The smallest variation from the line indicates low error and strong model fit.

**Figure 18:**
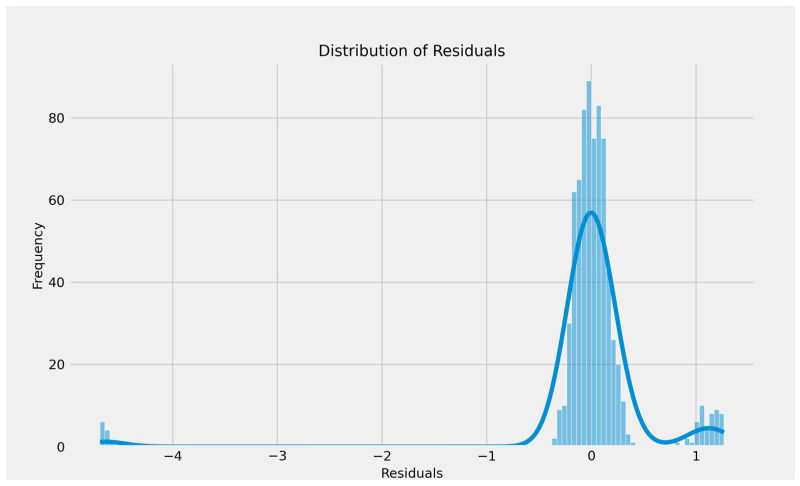
The distribution residuals for linear regression model.

The residual distribution is nearly centered at around zero and appears to be fairly normally distributed, supporting the assumption of homoscedasticity and implying that the linear regression model’s errors are unbiased. The little asymmetry and small clusters of outliers on both ends might suggest minor inaccurate modeling or a few unusual observations, but the general pattern shows that the model fits the data well (Figure 18).

**Figure 19:**
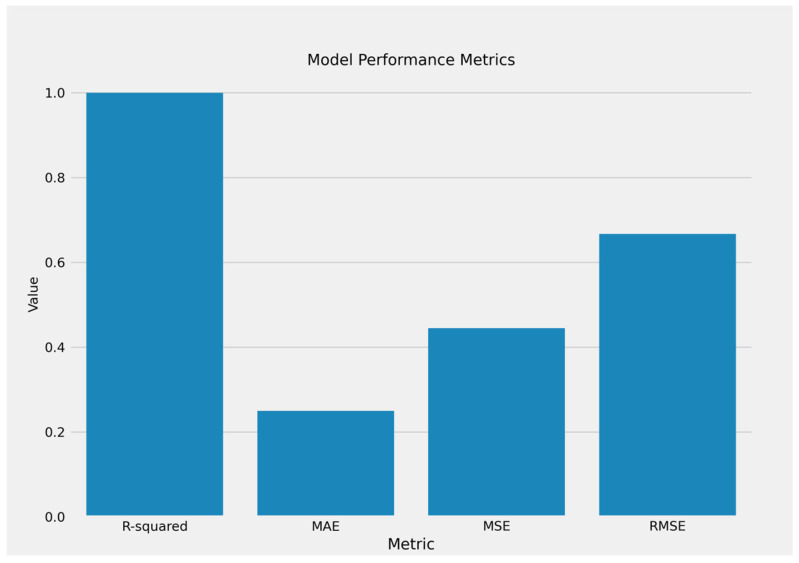
The performance metrics for linear regression model.

The bar chart of model performance measures shows that the linear regression model outperforms expectations. The R-squared value is at its peak of 1.0, indicating that the model explains nearly all of the variability in the dependent variable. The comparatively low MAE (Mean Absolute Error), MSE (Mean Squared Error), and RMSE (Root Mean Squared Error) values demonstrate that prediction errors are negligible, which reinforces the model’s reliability and accuracy (Figure 19).

**Figure 20:**
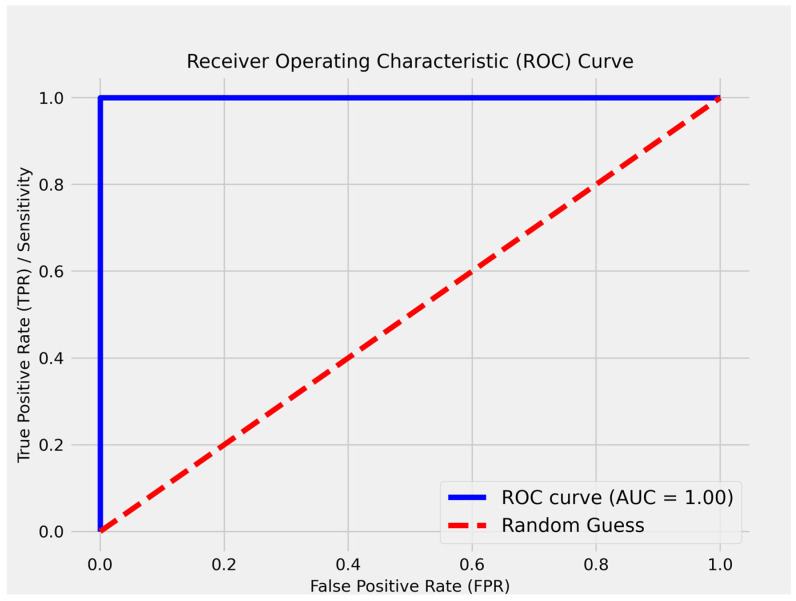
The ROC for logistic regression model.

According to Figure 20, the logistic regression model achieved the best possible prediction method, resulting in a point in the upper left corner or coordinate (0,1) of the ROC space, representing 100% sensitivity (no false negatives) and 100% specificity (no false positives), indicating a perfect classification. A random estimate would result in a point along a diagonal line (the *line of no discrimination*) from the bottom left to the top right corners (independent of the positive and negative base rates).

**Figure 21:**
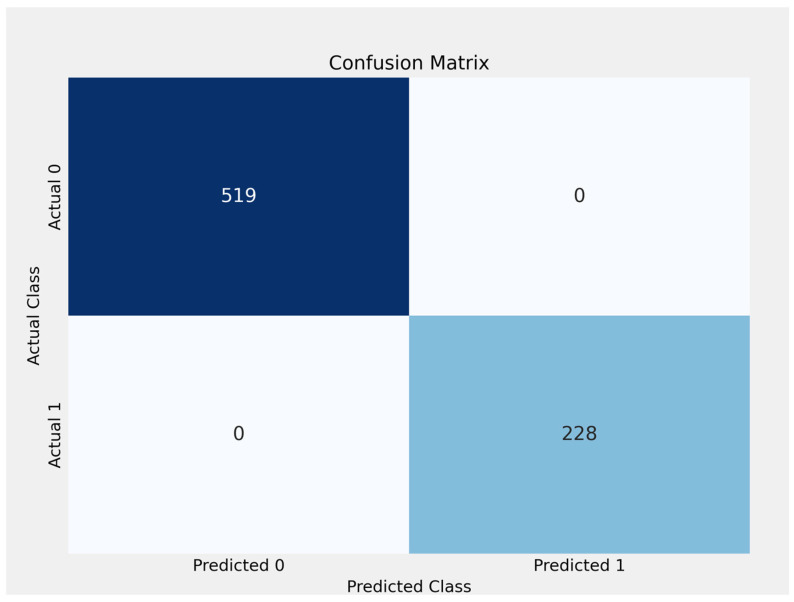
The confusion matrix for logistic regression model.

The confusion matrix demonstrates that the logistic regression model performed perfectly on the dataset, with 519 true negatives, 228 true positives, and no false positives or false negatives. This means that the model successfully predicted every instance of class 0 (cost < median) and class 1 (*Is_High_Cost* when cost > median), yielding 100% accuracy, precision, recall, and F1 score for both classes (Figure 21).

## 4. Discussion

The purpose of this study was to evaluate the possible cost value of various patients’ bills for surgery, chemotherapy, radiation, and radiotherapy for cervical cancer. Precise cost estimation for therapeutic interventions provides a valuable overview of the disease’s spread and post-treatment evolution, which is essential for clinical practice. It guides the choice of adjuvant therapy and enhances patient outcomes, but it also suggests swift action to prevent the disease’s severe effects on life expectancy and quality of life [66].

The necessity for accurate prognostic methods and early identification to lower expenses and increase patient survival rates, as well as to raise the screening lowering age and HPV vaccination rates for both males and females, is highlighted by the fact that disease progression might differ dramatically amongst individuals with identical clinical characteristics [67,68,69].

We want to highlight the advantages of using AI in pathology, including improved diagnostic accuracy, better efficiency in identifying cancer in its early stages, standardization and consistency, and the development of particular expertise [70,71,72,73].

These tumors have a worse prognosis, primarily because of their associations with other tumor types, which make staging more challenging and raise the cost of late identification [74,75].

Nevertheless, these tumors have the most successful therapy; patients respond well to the tumors’ surgical excision, and their survival rates range from 60–80% in the late stages to 100% in the early stages. Cervical cancer is a harmful disorder that can affect a patient’s health. However, paraclinical examinations may not provide a clear picture and may lead to the suspicion of cervical endometriosis rather than cervical cancer [64,65,66,67,68,69,76,77,78,79,80,81].

Cost and quality concerns are the Romanian healthcare system’s primary challenges. These costs pertain to inadequate funding and inefficient use of these assets. Informal payments, such as cash given to physicians and nurses in exchange for quicker care, can also cause issues as they skew people’s access to healthcare in a fair way [2].

Prevention needs to be prioritized, and the next logical step would be to investigate epigenetics. Furthermore, therapy in reference centers and multidisciplinary settings is beneficial [82].

Experience from developed countries has demonstrated that vaccination and screening programs are cost-effective and successful ways to prevent cervical cancer [83].

However, Romania has a shortage of skilled labor and infrastructure, which presents a financial obstacle and raises concerns about the viability of the health system in implementing these preventative measures.

As illustrated in Figure 3, the medical teams that specialize in the diagnosis and treatment of specific diseases have experienced a gradual disintegration over the course of nearly two years during the COVID-19 pandemic, particularly in the area of planned surgical treatment procedures. The pandemic was not an opportune moment for the implementation of new screening programs, as many medical facilities were closed, a situation corroborated by numerous other studies [84]. This reality may also have impacted the reported cases during that period. But even in this situation, in our study, cervical cancer incidence and costs are high due to a large number of cases recorded in the previous five years, according to data from Romanian cancer registries, Oncohelp, and DSP.

In Romania, a national initiative exists that offers a complimentary anti-HPV vaccine via family doctors. The topic of HPV vaccination remains highly contentious in the country, as it is neither mandatory nor included in the National Vaccination Program. The HPV vaccination initiative was first launched in 2008, targeting girls aged 10 to 11, yet only 2.57% of the eligible population received the vaccine at that time. In 2009, the age threshold was raised to 14 years; however, the program was halted in 2010 due to insufficient participation. Beginning in January 2021, the program was reinstated in a revised format for girls aged 11 to 14, and as of September 2021, the eligibility age was expanded to include those up to 18 years old. Consequently, HPV vaccination is currently available free of charge upon request at family doctor offices for girls aged 11 to 18 in Romania. Other segments of the female population and male adolescents do not qualify for complimentary vaccination through this initiative and must cover the full cost if they wish to receive the vaccine. The total expense for three doses of the Gardasil-9 HPV vaccine corresponds to the present minimum wage for in-pay employment [85].

While the Ministry of Health advocates for the vaccination of boys, it fails to ensure gender-neutral access to the vaccine. This oversight persists despite prior research demonstrating the advantages of the HPV vaccine, particularly in its nonavalent formulation, for the prevention of HPV-related illnesses and the reduction in both low- and high-grade cervical intraepithelial lesions among men [86].

A recent study examining the disparities in screening and HPV vaccination initiatives, as well as their effects on cervical cancer statistics in Romania, reveals that our national programs are hindered by complex procedures, insufficient funding, and inadequate motivation for healthcare professionals. Furthermore, the absence of information available to the eligible population contributes to a significantly low rate of screening and vaccination among women [87]. We conclude that the Romanian Ministry of Health must urgently undertake substantial awareness campaigns, implement strategies to enhance the functionality of these programs, and ensure consistent funding.

In Romania, vaccination is also a very cost-effective way to prevent cervical cancer. Over time, it appears to be a more cost-effective approach to immunize teenage females against HPV and screen them for the virus using a visual inspection with acetic acid between the ages of 30 and 65. This may be performed every five or ten years [88].

Strategies that resulted in fewer follow-up visits and better follow-up testing and treatment were the most cost-effective ones. Using a one- or two-visit screening strategy that included visual inspection of the cervix with acetic acid or DNA testing for human HPV in cervical cell samples, screening women once in their lifetime at the age of 35, reduced the lifetime risk of cancer by about 25 to 36 percent and saved less than $500 per year [89].

The international scientific community is enthusiastic about the uncounted perspectives that are taking shape through the introduction of AI in the treatment algorithm of neoplastic pathology, especially in metastatic forms, whose therapeutic perspectives are limited by the classical surgical approach [90].

Also, AI’s increased applicability is represented by its introduction into the diagnostic and treatment algorithm of very rare cases of malignant transformation of adenomyosis, a situation in which the relatively limited experience of practitioners is complemented by AI’s indisputable advantages [91].

Studies analyzed the overall costs of several screening procedures (Pap smear alone, HPV + Pap, co-testing) and found that the highest price is frequently treatment for high-grade lesions or cancer itself [92]. Linear regression can show how changes in screening intervals, test types, or patient characteristics affect the total cost. Cervical cancer analysis is possible with this method, such as calculating the financial effects of a drop in test costs or a shift in patient compliance [93,94]. In cervical cancer screening research, both logistic regression and linear regression models are often employed and exhibit strong performance for their respective uses [95]. In cervical cancer research, logistic regression models have been effectively applied to classify results and find important predictors of high-cost cases or cost-effectiveness, frequently attaining high sensitivity and accuracy [96].

Combining logistic and linear regression enables a thorough analysis: logistic regression classifies situations as cost-effective or not based on thresholds such as the incremental cost-effectiveness ratio, while linear regression forecasts real costs [94].

The cost-effectiveness ratios of screening programs, which are very sensitive to the number of Pap smears provided throughout a lifetime, are not the basis for the variability in screening practices among high-income nations [97]. However, there are situations, such as dysgerminomas, where there is no significant precedent from a statistical perspective, and the calculation of this ratio is complex to achieve [82].

Implementing AI in genetic research could contribute equally to improving the cost-effectiveness ratio to optimize the large-scale projects carried out in this field [98].

Prior research had determined preventive cryotherapy to be an affordable cervical cancer intervention for low- and middle-income nations [99].

Public health professionals must also closely monitor coverage, compliance, resource utilization, and outcome factors to spot problems and areas that require attention. Models will always surpass true-life evaluation, and our model may be improved and updated as more data come in [100].

Restructuring the Romanian cervical cancer screening program for cost-efficiency makes sense when it comes to bringing services closer to females. It is essential to carefully analyze the various policy factors of this development, including the demand for human resources, the interests of all parties involved, organizational characteristics, and the objective population’s approach.

### Limitations of the Study

Our study has some limitations. First, although costs are examined over a period of several years, the study does not specify whether monetary values have been adjusted for inflation. This lack of adjustment limits the comparability of costs over time and may influence conclusions about cost trends. Second, the scope of the analysis is limited to Western Romania. As a result, the conclusions may not be applicable to other areas of the country or to health systems characterized by different infrastructure, demographics, or resource availability. Third, the data include years affected by the COVID-19 pandemic, which disrupted standard screening and treatment services. As a result, this may distort trends in incidence, costs, and treatment during this period. Furthermore, although the AI-based forecasting and regression models demonstrate performance with the existing dataset, their applicability to new data or different medical contexts may be limited without external validation.

## 5. Conclusions

Our goal was to give policymakers quantitative and qualitative information on the trade-offs between various screening approaches that use new screening technologies concerning HPV vaccination. We emphasize the significance of alternate screening approaches that depend on age and immunization history.

While logistic regression is appropriate for determining if costs are over or below a cost-effectiveness threshold, linear regression is useful for forecasting the ongoing costs of cervical cancer screening and treatment. Both models are essential to cost-effectiveness studies because they allow for advanced evaluation and promote evidence-based medical decisions for the therapy and prevention of cervical cancer.

We also highlighted the costs that would increase as a result of cervical cancer if further steps were not taken to stop the disease’s aggressive and quick spread among women of all ages. It has been noted that these costs are growing linearly, resulting in patients’ quality of life being negatively impacted.

## Figures and Tables

**Table 1:** Descriptive statistics: cost–stage.

	Cost (€)									
	In Situ	IA	IB	IIA	IIB	IIIA	IIIB	IIIC	IVA	IVB
No.	11	97	345	256	865	337	554	378	351	538
Mean	218.00	1570.78	1859.52	7675.36	7425.57	7757.38	7999.28	7977.82	3914.00	330.00
Std. Deviation	0.00	323.89	203.21	1142.49	1367.77	1470.06	1171.35	1203	0.00	0.00
Minimum	218.00	218.00	1450.00	4711.20	4711.20	4155.6	4155.6	4155.6	3914.00	330.00
Maximum	218.00	1668.00	1961.20	8114.00	8114.00	8355.6	8355.6	8355.6	3914.00	330.00

**Table 2:** Cost–treatment distribution.

Stage and Procedure	Number of Patients	Cost of Treatment (Euro)/Patient
In Situ—Conization	11	218
Stage IA—under or equal to 45 years—Conization	5	218
Stage IA—under or equal to 45 years—Surgery	10	1450
Stage IA—over 45 years—Conization + HT	82	1668
Stage IB—Surgery	68	1450
Stage IB—Surgery, chemotherapy, brachytherapy	277	1961.2
Stage IIA—Surgery, chemotherapy, brachytherapy	33	4711.2
Stage IIA—Surgery, chemotherapy, radiotherapy	223	8114
Stage IIB—Surgery, chemotherapy, brachytherapy	175	4711.2
Stage IIB—Surgery, chemotherapy, radiotherapy	690	8114
Stage IIIA—Surgery, chemotherapy, radiotherapy, brachytherapy	289	8355.6
Stage IIIA—Chemotherapy, radiotherapy, brachytherapy	48	4155.6
Stage IIIB—Surgery, chemotherapy, radiotherapy, brachytherapy	507	8355.6
Stage IIIB—Chemotherapy, radiotherapy, brachytherapy	47	4155.6
Stage IIIC—Surgery, chemotherapy, radiotherapy, brachytherapy	344	8355.6
Stage IIIC—Chemotherapy, radiotherapy, brachytherapy	34	4155.6
Stage IVA—Chemotherapy, radiotherapy	351	3914
Stage IVB—Chemotherapy	538	330

**Table 3:** Descriptive statistics: age–stages.

	Age									
	0	IA	IB	IIA	IIB	IIIA	IIIB	IIIC	IVA	IVB
No.	11	97	345	256	865	337	554	378	351	538
Mean	55.82	55.63	57.04	59.32	57.38	62.77	58.37	56.27	61.82	58.82
Std. Deviation	5.09	9.48	10.30	11.79	11.35	8.86	10.09	10.56	8.39	12.08
Minimum	49.00	30.00	26.00	33.00	29.00	33.00	32.00	34.00	33.00	32.00
Maximum	67.00	71.00	84.00	88.00	88.00	89.00	87.00	86.00	84.00	84.00

## Data Availability

The data presented in this study are available upon request from the corresponding author.
